# Work and “Mass Personal” Communication as Means of Navigating Nutrition and Exercise Concerns in an Online Cancer Community

**DOI:** 10.2196/jmir.2594

**Published:** 2013-05-31

**Authors:** Brad Love, Charee M. Thompson, Brittani Crook, Erin Donovan-Kicken

**Affiliations:** ^1^Belo Center for New MediaDepartment of Advertising and Public RelationsUniversity of TexasAustin, TXUnited States; ^2^School of Communication StudiesOhio UniversityAthens, OHUnited States; ^3^College of CommunicationDepartment of Communication StudiesUniversity of TexasAustin, TXUnited States

**Keywords:** technology, young adult, psychosocial factors, social support, cancer, communication, exercise, dietetics, Internet

## Abstract

**Background:**

Health and psychosocial outcomes for young adults affected by cancer have improved only minimally in decades, partially due to a lack of relevant support and information. Given significant unmet needs involving nutrition and exercise, it is important to understand how this audience handles information about food and fitness in managing their cancer experiences.

**Objective:**

Using the theory of illness trajectories as a framework, we explored how four lines of work associated with living with a chronic illness such as cancer (illness, everyday life, biographical, and the recently explicated construct of communication work) impacts and is impacted by nutrition and exercise concerns.

**Methods:**

Following a search to extract all nutrition- and exercise-related content from the prior 3 years (January 2008 to February 2011), a sample of more than 1000 posts from an online support community for young adults affected by cancer were qualitatively analyzed employing iterative, constant comparison techniques. Sensitized by illness trajectory research and related concepts, 3 coders worked over 4 months to examine the English-language, de-identified text files of content.

**Results:**

An analysis of discussion board threads in an online community for young adults dealing with cancer shows that nutrition and exercise needs affect the young adults’ illness trajectories, including their management of illness, everyday life, biographical, and communication work. Furthermore, this paper helps validate development of the “communication work” variable, explores the “mass personal” interplay of mediated and interpersonal communication channels, and expands illness trajectory work to a younger demographic than investigated in prior research.

**Conclusions:**

Applying the valuable concepts of illness, everyday life, biographical, and communication work provides a more nuanced understanding of how young adults affected by cancer handle exercise and nutrition needs. This knowledge can help provide support and interventional guidance for the well-documented psychosocial challenges particular to this demographic as they manage the adversities inherent in a young adult cancer diagnosis. The research also helps explain how these young adults meet communication needs in a “mass personal” way that employs multiple communication channels to meet goals and thus might be more effectively reached in a digital world.

## Introduction

Despite advances in medical care and treatment, survival rates for many young adults affected by cancer have not improved in recent decades [1,2]. Posttreatment issues remain common [3], in part from reduced access to age-appropriate support resources, which can negatively affect psychological and social development [4-8]. Additionally, a lack of information about exercise and nutrition is acutely prominent during the young adult cancer journey [5,9], despite exercise and nutrition’s role in managing symptoms and contributing to health outcomes.

As many as 52% of adolescent cancer survivors do not engage in enough physical activity, and 79% do not meet guidelines for fruit and vegetable consumption [10]. Poor diet and exercise behaviors contribute to comorbidities, including secondary cancers, cardiovascular disease, and diabetes (see Arroyave and colleagues [11], for a review). Furthermore, unmet physical and daily living needs are related to greater depression and anxiety [9].

An individual’s illness trajectory encompasses more than the physiological course of the illness; it involves broader and more social factors such as work associated with the illness, the impact on relationships, and how these factors affect outcomes for the person diagnosed with the illness [12]. Serious illnesses such as cancer are experienced as trajectories, “the course of an illness over time, plus the action of clients, families, and healthcare professionals to manage the course” p. 257 [13], which involves negotiating types of work to manage the broader challenges associated with disease [13]. Identifying how the young adult cancer community handles nutrition- and exercise-related work during their cancer trajectory would contribute to understanding the development of a “new normal.”

Additionally, exploring a topic such as nutrition and exercise that has relevance across cancers presents an opportunity to learn about the broader young adult cancer experience. Cancer as a disease consists of many different conditions. As such, cancer research tends to focus on specific diagnoses instead of examining the overall experience; thus, another benefit of research focusing on broadly useful topics such as food and exercise is the chance to better understand commonalities among cancer experiences.

### The Work of Establishing a New Normal

A cancer diagnosis disrupts every facet of life and introduces chaos as a normal part of daily existence [14], both for those receiving the diagnosis and their supporters. As a result, cancer becomes more than a biological and physical state—it becomes a social experience [15], and for those affected, managing illness necessitates additional tasks including learning to access needed information, re-negotiating identities and relationships, and coping across the illness’ effects [16,17]. These efforts help patients adjust to life after diagnosis and define a “new normal” as they move through the illness journey [18].

An informative way to understand this time of transition is Corbin and Strauss’s [13] theory of illness trajectories with its central component of work. Their examination of couples dealing with chronic conditions specified three lines of work: illness, everyday life, and biographical. Illness work consists of managing tasks related to the health condition, ranging from regular responsibilities such as tracking blood sugar to crisis prevention. Everyday life work covers tasks related to maintaining a functioning household such as housekeeping, child-raising, marital obligations, and occupational responsibilities. Biographical work involves management of the biographical disruption that comes with chronic illness, including the reconstruction of identity and life planning [13].

Based on Corbin and Strauss’s analysis, Donovan-Kicken and colleagues [15] summarized a number of dimensions that characterize cancer management behaviors as work, including completing tasks and assigning responsibility for completion; exerting effort, allocating resources, coordinating duties; and dividing up labor throughout the trajectory. Further, Donovan-Kicken and colleagues identified communication work as an important fourth line of work to consider in understanding illness trajectories. Communication work entails the coordinated management of creating, exchanging, and interpreting messages pertinent to the experience of illness and is embedded in the larger context of daily life and ongoing relationships [19]. Because it constitutes the most recent extension of the theory of illness trajectories, we provide an additional overview of the construct of communication work next.

As has been noted in a range of chronic-illness environments (eg, [20,21]), exchanging information about a disease may occur as demanding, challenging work beyond simply seeking and receiving information to meet a need [22]. Communication work reflects an updated way of thinking about communication in illness trajectory research [19]. Studying communication work “means shifting the focus from communication as a necessary behind-the-scenes duty that allows the other lines of work to exist and succeed, to a focus on communication in the foreground, wherein the processes of exchanging messages and achieving meaning are examined as essential elements of the illness experience” p. 648 [15]. Because the construct of communication work concerns the labor of interacting with information and with other people, it provides a means of conceptualizing the effort that people devote to locating, using, understanding, sharing, and managing information both intra- and interpersonally [15,21]. It enables scholars to account for the burden, effort, process, resources, and outcomes of communication in addition to how these aspects relate to illness, everyday life, and biographical work.

Articulation work refers to the coordination activities necessary to complete all four lines of work (illness, everyday, biographical, communication) and thus sits across the illness trajectory framework, playing a role in each aspect [19]. Articulation work “is work that gets things back ‘on track’ in the face of the unexpected,” p. 275 [23], encompassing the organization necessary to make arrangements for other work to take place [20]. Such efforts play an essential role in the management of work flow, according to Strauss [24], as articulation is “the coordination of lines of work. This is accomplished by means of the interactional processes of working out and carrying through of work-related arrangements” p. 87 [25].

In explicating communication work, Donovan-Kicken analyzed the properties of two existing elements of the theory of illness trajectories: information work and articulation work [19]. The three related concepts intermingle in that information work, a functional activity about searching for information, fits inside the more comprehensive concept of communication work, which also entails the interpersonal communicative efforts not discussed either as part of or in service to the other three lines of work. While communication involves gathering information, it also entails effortfully employing that information to interact with other people and the work required to negotiate relationships, including expressing emotions and moods, which can be particularly demanding for people dealing with illnesses or disabilities [26].

Previous research on communication work in the cancer context has indicated that communication work is present in social and personal relationships as people engage in effortful tasks such as disclosing illness status, explaining medical details, defending treatment decisions, and communicating support [15]. Some of those interactions entail traditional information work in making sense of illness-related information like risks and success rates for treatment options. Other interactions go beyond that scope into what is labeled here as communication work and involve a broader array of interpersonal obligations and conversational demands demonstrated in prior research, such as maintaining social networks, managing private information, and comforting others [27-30]. Isolation and uncertainty serve to make these work tasks even more labor-intensive [31]. Further, patients and their support networks may engage in articulation work to coordinate communication efforts and to divide up the labor, for example by assigning information dissemination tasks [15].

### Work in the Young Adult Cancer Context

Young adults with cancer are likely to experience the four types of work as uniquely challenging. Consistent with research on “emerging adulthood” [32], the transition from adolescence to adulthood (18 to 39-year-olds, per the official US Department of Health and Human Services definition) [33] is a unique time for young adults critical to future well-being [2,34]. The experience of cancer derails young adult identity development and in many cases makes achieving milestones (eg, attending college, getting married) more difficult [35-37].

The well-being of those affected by cancer depends in part on psychological and social development [38], and many young adult patients deal with serious psychosocial troubles [6,39-41]. Even those doing quite well still deal with quality-of-life concerns ranging from worries about how their health impacts family members to reduced marriage and fertility rates [42-44]. The mobile nature of young adult lives can worsen unmet support needs and further limit contact with health care providers [45] as they transition among cities, jobs, and medical options. Despite these issues, researchers lump young adults in with other demographics and fail to examine their age-specific needs [4,8].

In general, young adult cancer survivors experience significant stress around information needs [2]. While almost every respondent to one broad survey (N=879) expressed a need for further information about illness and treatment, two-thirds voiced a desire for content about exercise and nutrition specifically [2], findings supported by other projects [7,8,46]. This scholarship suggests that the young adult cancer community has needs that are different from the general cancer community, including needs related to information about exercise and nutrition. To better understand that trajectory, we posed the following research question:

RQ1: What is the nature of work around exercise and nutrition for young adults affected by cancer?

### Mediated Communication and Work

Young adults live digital lives [47,48], and those affected by cancer are no exception [49,50]. Health professionals and patient advocates increasingly recognize digital media for spreading health information tailored to youth [51-56]. Thus, not only does studying mediated communication provide an opportunity to learn more about the exercise- and nutrition-related needs and behaviors of young adults with cancer, but the online context also presents an innovative approach to applying the concept of communication work and the theory of illness trajectories.

Corbin [57] acknowledges that theory of illness trajectories could and should be updated to consider modern developments. When Corbin and Strauss [13] developed illness trajectories, the impact of technology was yet to be realized for its informative and supportive utility in health contexts. Further, Donovan-Kicken and colleagues’ [15] definition of communication work also supports the utility of digital media for its ability to help individuals manage and process information [58] through interactions with others sharing similar health concerns and information needs [49,59]. We felt it important to recognize the influence of digital media as a primary source of information and support for young adults dealing with cancer [50], as understanding choices and tailoring to the needs of young adults can allow for effective and efficient digital support interventions [53]. Thus, we posed the second research question:

RQ2: What is the role of mediated communication in illness-trajectories work among young adults affected by cancer?

## Methods

### Sample

Data from an online young adult cancer forum were compiled from de-identified text-only files of full conversation threads, after Institutional Review Board approval and cooperation from community managers. Community membership registration states that content may be used for research purposes as well as a statement that content can be used for “well-behaved,” noncommercial efforts, subject to the definition and discretion of network administrators.

The forum is one of several online, English-language forums open to any young adult affected by cancer across the treatment spectrum. The online community that produced the text has several thousand users responsible for almost 20,000 monthly visits. Based upon required questions posed to all new members and the content of the community discussions, we can infer that community members are within an age range of 18-39, fitting the generally accepted AYA definition. Additionally, user data aggregated by the community indicates that users are slightly more likely to be female, have English as a mother tongue, and come primarily from the United States with single-digit percentages of members participating from Australia, the United Kingdom, and Canada.

Posters in the sample discussed cancer as a lived, first-person experience as opposed to writing about others’ happenings. Care providers and supporters of individuals diagnosed with cancer do make up a small percentage of the community membership but did not come up in this sampling.

The first 3 authors (hereafter referred to as researchers) read through a subsection of the entire pool of threads to identify relevant nutrition- and exercise-related terms, including exercise, sport, work out, gym, nutrition, nutrients, diet, and weight management. Researchers compiled and agreed upon a list of terms and then extracted all other threads in which at least one of the terms was discussed. The original post did not have to be about nutrition or exercise to be included in the sample. Often, individuals would mention nutrition and/or exercise in response to the original comment that may have had nothing to do with nutrition or exercise; however, it was clear that nutrition and exercise were ways individuals understood and responded to others’ questions and concerns. This way, we were able to see what nutrition and exercise mean to people affected by cancer in a naturalistic way that is more in line with how individuals experience illness trajectories.

The final text sample consisted of 1000 posts comprising 79 complete threads (initial post and corresponding comments) across 342 pages. Threads were initiated by users from January 2008 to February 2011 and ranged widely in length and duration. Some threads received little engagement, consisting of only an initial post. Other threads addressed engaging topics and continued for more than a hundred follow-up posts during the course of 2 full years.

### Data Analysis

We employed an iterative approach over 4 months to analyze the text. We grounded our analysis in sensitizing concepts [60] from Corbin and Strauss’s [13] scholarship on work and illness trajectories, along with Donovan-Kicken et al’s [15] review and contribution, as well as Becker’s [14] phenomenological research on chaos and sense-making during life crises. Chaos and uncertainty research presents important concepts in this context because they signal the work of creating a “new normal” for individuals dealing with difficult life experiences such as a cancer diagnosis. Engaging these texts allowed the research team to better understand the relationships among work, uncertainty, and daily life for those affected by illness. By examining the data initially and reviewing it following sensitization to key concepts, researchers operated per grounded theory expectations of continuous data collection and analysis [61].

Using constant comparison methods [62], we looked for themes in the text to better understand the role of exercise and nutrition in the young adult cancer experience. Researchers examined the text incrementally to allow for intermittent discussion of emergent themes so that ideas could be tested and improved during follow-up readings [63,64]. All researchers contributed to guiding the data analysis by analyzing text individually and then discussing and challenging findings as a group. The research team established coder agreement through discussions over 4 months and based findings on agreement that the themes and relationships were present [65].

The team maintained theoretical notes throughout the coding process and used peer debriefing to establish trustworthiness of findings [66]. Through regular debriefing, the fourth author served to verify the researchers’ application of the key concepts to the data and provided a disinterested perspective during analytic sessions. The following section presents findings and exemplars. All proper nouns such as names of people and treatment facilities are pseudonyms, and potentially identifying details are edited for anonymity.

## Results

Our analysis of the data suggests that managing cancer in the context of nutrition and exercise is effortful, demanding, and burdensome in ways consistent with Corbin and Strauss’s [13] conceptualization of work. These lines of work—illness, everyday life, biographical, and communication—overlap and compete as young adults try to achieve some semblance of normality. Our findings suggest that not only do eating and exercising become more effortful, but they also make other types of work more onerous. Each line of work is elaborated next to address our first research question concerning the nature of work around exercise and nutrition for young adults affected by cancer.

### Illness Work

Illness work refers to the scope of tasks connected to managing the health condition, including both daily responsibilities such as taking pills and irregular activity such as follow-up visits with an oncologist. Illness work is most prominent in our data concerning how nutrition and activity can be employed as a way for these young adults to maximize their health by fighting cancer itself or managing the side effects of cancer and treatment.

Young adults in our sample describe how modifications in diet and exercise can maximize health during treatment by serving as another means of combatting the disease. One member explains: “I was on a high-dose chemo regimen for CNS lymphoma (brain tumor), so for me it was good to have high protein and lots of fresh fruits and veggies, according to what my dietitian said. My docs said I couldn’t have supplements because of my treatment.”

In addition, young adults describe how nutrition and exercise become ways of managing side effects. They talk about being surprised and disheartened by the way cancer affected their bodies—through weight gain and loss, changes in body composition or mobility (eg, loss of a limb), and decreases in energy. As one member wrote:

While I was going through the chemo, I did not realize what I do now, that prednisone and like drugs mess with your body’s sugar metabolism. So, now when I am on it for a month at a time, I find I can minimize the weight gain by watching my sugar intake. I still gain a bit (5-8 lbs) overnight it seems, and get the moon face and fat neck, but the overall number of pounds is much less that when I did not watch my sugars.

Making diet modifications and exercising become ways of combating and alleviating negative effects while asserting some control. As one poster succinctly commented, “at least during treatment you feel like you’re actively doing something about the cancer.”

Exercise, in particular, is described by young adults as mandatory because of its role in weight management and importance in adjusting to physical changes from treatment. Posters document their activity regimens and successful outcomes as necessary illness-driven aspects of their trajectories. One member wrote about his daily gym routine:

With me it was pretty quick after I finished radiation treatments that I got back to it. I just started off easy and upped time/difficulty every day. I gave myself a week to rest before I got back to it. For me it wasn’t an option not to exercise because I’ll backslide at an amazing rate due to nerve damage. Fun stuff. I don’t know. For me, I just tried to mentally psych myself up for it. And told myself I HAVE to do it.

Physical changes often take the form of working against such nerve damage or adjusting to amputations. A poster discussed the consistent work required to re-establish hand mobility following surgery:

I have had to have physical therapy in order to learn how to use my right hand again as I had a Ewing’s Sarcoma of the left middle finger. My finger was amputated and the forefinger pulled towards the other fingers in a procedure called a Ray Resection. I was determined and within 4 months of surgery had almost full use of that hand again. People don’t even notice I missing something until I show them.

These exercise and nutrition efforts constitute illness work because fighting cancer and managing side effects are aspects of dealing with illness-related issues above and beyond basic tasks of normal life. In dealing with how cancer has affected their bodies, consistent activity and a focus on food become obligatory tasks for these young adults to deal with their illnesses and accompanying issues. Exercise and nutrition offer a way of grappling with the weight changes common to cancer treatment as well as learning to function with more extreme physical issues such as nerve damage or balance problems.

### Everyday Life Work

According to Corbin and Strauss’s [13] original conceptualization, everyday life work involves activities done to maintain a household and life as an independent adult. While diet and exercise may not be as crucial to household functioning as paying bills, young adult comments suggest depleted energy and physical (in)capacity impact the feasibility of everyday tasks. As one contributor wrote, “Time is pushing down on me and it is going to run out. Every day I think, ‘I don’t have enough time!’ while I am simultaneously washing my dishes, having a phone conversation and cooking dinner!”

Our reading of the data suggests that nutrition and exercise activities make the everyday life work of household management more effortful and onerous. For example, preparing meals and exercising become “work”, and young adults talk about not fully appreciating food and fitness as leisure. Diet and exercise concerns move from being issues of “want to” to “have to”. Cancer takes away choices individuals have with respect to diet and exercise. As one young adult describes:

I’m tired of being virtuous. I’m tired of doing an hour of meditation. Of making perfect organic meals three times a day. Of making a pint of fresh carrot juice. Of drinking green tea instead of a nice cup of coffee. Of missing desserts. Of the possibility of getting drunk (since I know it will depress my immune system). Of exercising even when I feel bad. Of reading dozens of self-help books. Of feeling I should be doing something productive like guided imagery when I lay down to rest. Of acupuncture appointments. Of the uphill task of researching supplements and making intelligent decisions about dosages.

For this community member, dealing with food and activity adds to the burden of the illness trajectory by creating demands to make life as an independent adult that much harder. Activities that may have been enjoyable pre-cancer (ie, preparing food, exercising) switch to effortful parts of life.

Required everyday nutrition management plays into difficult feelings, as food preparation creates burdensome demands per treatment or maintenance requirements, according to many young adults. The low-iodine diet recommended to thyroid cancer patients stands out as an example of nutrition management becoming everyday life work and driving strong emotions: “But that combo of deprivation and exhaustion and effort required to eat in an iodine-less way is a killer. It sucks. I made the mistake of going out to eat once…It was so hard not to cry into my lettuce with olive oil and vinegar (and to not grab fistfuls of French fries and oysters off the table)!! That was torture.”

Additional effort and time for nutritional needs adds to the burden of the young adult cancer experience, particularly intersecting with life stage issues such as being away from family or having extensive early career work burdens. As one member wrote when discussing her low-iodine diet:

I can’t believe I have to do this for another nine days. I cannot cook anymore! I spent an entire afternoon making my tortillas for this last week and I don’t have the time! I’m a single woman with a full time job and no friends nearby. It’s like the whole thing was created for people with the time to cook and/or the money to purchase organic meats so they won’t starve. It’s bullshit!...I’m so over this and so angry that I want to call my endocrinologist and bitch, just so I can blame someone and because it concerns me that I feel so physically bad. I don’t know what I can do to fix this. Take sick days so I can cook?? I am at the end of my rope. I’m tired, pissed off, really hungry, and out of patience.

Exercise and nutrition impact everyday life work by forcing previously optional activities to become required ones. Physical activity and food management become necessary household responsibilities essential to well-being and ability to complete other work. Previously positive or interesting hobbies can even transition from blessings into burdens.

### Biographical Work

Exercise and nutrition affect biographical work by influencing how individuals process temporal aspects of identity re-construction, particularly when viewed through pre-cancer experiences. Fitness and food affect how young adults view their pre-cancer selves, how they deal with the present, and how they prepare for the future. Biographical work is particularly colored by body image perceptions—and resulting self-esteem attitudes—in addition to concerns about making the most of life through improved nutrition and exercise; thus, the topic areas become ways of adding hope to their identity re-building.

Comparison to prior fitness achievements and pre-cancer selves presents a direct challenge to aspects of identity re-construction including making sense of ongoing effects. As one member wrote:

I am a choreographer and was in the studio dancing 5 or 6 days a week before I was diagnosed. I feel like cancer did a number on that part of my identity. For me it was not just a matter of having the physical energy to get back into it, there has been a huge mental block there too.

Viewing the present through an activity-related past adds an identity-driven challenge inherent in any diagnosis and treatment regimen; many young adults no longer know how to label themselves in line with their exercise efforts nor how permanent those classifications are. As one post read: “I was an athlete before one of many surgeries. I hate using ‘was’. I suppose it is now in dormancy…How do you guys deal with temporary or permanent loss of sports?” Young adults in such situations are then forced to re-evaluate key self-ascribed labels (eg, “athlete”) that may have been foundational pre-diagnosis, and in many cases, finding new labels is a monumental challenge for which they are unprepared.

Comparisons to prior exercise routines also overlap with key moments in personal relationships and major events that helped individuals mark time in their pre-cancer lives. Changing fitness norms can impact regular routines as well as milestones that serve as personal highlights and create identity within families. One member commented regarding difficulties with re-establishing his exercise regimen and the impact on noteworthy fitness events related to his long-term identity as a cyclist:

Pre-cancer, I was at the gym three-four times a week, and was in a nice groove of getting up at 6 for a work-out before heading to the office. Needless to say, life’s a bit different now, I really love and need my rest, and I am as stiff as hell when I wake up in the morning. At the end of the day, I am tired from working all day!!…I really feel like I want to start - slowly - getting back into an exercise groove. But I am having a devil of a time kick-starting this thing. I am also an avid cyclist and have pretty much missed the whole riding season. In a few weeks, the annual MS 150 Ride which my partner and I have been doing for years is coming up, and I feel a certain sadness that I just didn’t have the strength or energy to train this year.

Along with identity questions driven by pre-diagnosis activity-focused experiences, exercise and nutrition also affect ongoing biographical work by influencing feelings about body image and self-esteem. For some, fitness and food are a means of recognizing and expressing how life is different post diagnosis. As one person noted: “I finished my chemo a little over two years ago, and am only just coming to grips with the changes. I am NOT the same person I was BC (before cancer). Lots has changed: I get tired a lot easier, my diet has changed (can’t eat fast food, gotta watch fat intake), my eyesight is different, and on and on.” Young adults in such situations grapple with the challenge of creating a new identity more in line the current reality, including what cancer has done to dietary habits and body composition. Each meal time, for example, can force ongoing realizations that life has irrevocably changed.

Exercise and nutrition can be empowering for other young adults, however, as they use weight management to assert control over an area of life and self-perception. One poster discussed her weight management as something she could track and take comfort in while moving forward from the difficulties of illness: “I lose about half to a kilogram a week but if it’s less (like this week 0.1) I’m not upset as I know it’s 0.1 that won’t be back again. The biggest thrill is—and I’m sure you’ll agree—walking in to shops and grabbing heaps of things you can try on and they look good.” This community member found empowerment through exercise- and nutrition-related changes and the ability to create progress in some area of her life.

A future focus is also a key theme of how exercise and nutrition influence young adult biographical work. Some members of the community discussed food choices, for example, as taking precautions and staying healthy. Food becomes a way of investing in their future selves, such as in this post: “My diet I have changed some things. I eat more veggies, asparagus especially, because I read/was told that it gets rid of bad cells, and dead cells. I have no idea if it does, but I figured can’t hurt to eat fresh veggies.” Food allows young adults such as this to take daily action with future well-being in mind and move beyond the extremely focused present required in treatment or the past-centered framework common to those struggling with identity re-creation [67].

Exercise activity can play a similar role, according to members, by creating situations allowing for personal growth and changed self-perceptions through programs such as First Descents, an outdoor adventure camp for young adults affected by cancer:

I have found more of myself with life after cancer by going to a young adult retreat by First Descents…You get to find life again by kayaking on the river…[k]ayaking made me realize if I can beat cancer, I can beat the rapids on the river. You see, one of my fears is drowning, and after flipping over after I hit my head on into a rock, I survived. I realized not to be so scared and to live my life. I will run those 5K’s, join a gym class, socialize more by inviting family members over more often.

Biographical work makes up a large portion of the exercise and nutrition discussions. Ongoing development of self-image intertwines with how young adults remain or regain activity as well as how they cook and eat, particularly in social situations. Factors including weight gain play a central role in how young adults view themselves and manage interactions, particularly when considered in light of their pre-cancer selves.

### Communication Work

Communication work describes the coordinated management of creating, exchanging, and interpreting messages, which affects whether individuals choose to communicate with others, share information, and use communication technology as a means of managing all types of work related to exercise and nutrition. The interplay of how users employ the mediated online community to discuss and prepare for face-to-face interpersonal conversations offers examples of how this specific demographic employs a combined “mass personal” approach to their communication work needs [68,69]. These findings help respond to our second research question about the role of mediated communication in illness-trajectories work among young adults affected by cancer.

An aspect of communication work discussed online is the challenge young adults feel in choosing to relate their cancer experience to others, particularly regarding diet restrictions. Discussing food and eating requires message preparation and planning for responses. According to the digital discussions, online groups and resources play a constructive role in helping individuals do the work needed to prepare for these offline conversations; participants exchange advice about communicating with doctors, loved ones, and work colleagues about food, in particular, and note the online community’s importance with comments such as, “Thanks for the response, sharing information does help us all.”

Similar communication work appears regarding exercise and nutrition in posts regarding message preparation for everything from medical-office visits to explaining post-treatment life to family members. Sometimes, communication work in this environment also entails trying to understand or predict reactions to communicative efforts about exercise, for example, including different responses among community members themselves. One young adult wrote to ask about a fellow survivor’s reaction to communicating about fitness issues, seeking help to understand his perspective as an initial step to effectively reach him:

He is mad that he lost his muscles, however is not really getting out to the gym to get them back. It’s as if he can’t see that it could be worse, he could be dead. I have tried talk to him and help him see that there are so many great things that he could learn from our experience with chemo and that he should appreciate the life that he does have but he just is not hearing me…can anyone help me to understand his point of view or how to help him because at this point I’m at a loss for words and it hurts my heart to see him wasting the life he has been given a second chance at away sitting inside all the time feeling sorry for himself and like his life is ruined. HELP!

For this community member, her digital peers were her preferred source for trying to understand past interpersonal conversations and also how to design messages most likely to be effective in future face-to-face interactions. The digital space became her testing ground for real-life discussion.

Community members in this case responded by offering thoughts on how to address the friend’s likely ongoing biographical work related to fitness and personal identity, including what kinds of messages would be likely to get him to open up about his illness trajectory. For example, “Guys tend to take a loss of strength and stamina a lot more seriously. Though I can say from personal experience that the whole loss of strength and stamina has been extremely difficult for me. I think guys take it as a blow to their manhood though.”

Pressure to effectively communicate one’s needs also means that some young adults decide to avoid social situations related to activity or food. For example, the necessary communication work involved in explaining requirements to others can mean avoiding public eating experiences, as one young adult stated, “I also find being forced changed [sic] to my eating really stressful. I don’t even like eating out because I feel out of control.” Not being in a food-based situation eliminates the need for difficult communication.

Digital technologies and the involved communication work also impact other types of work related to exercise and nutrition, in line with Corbin and Strauss’s [13] observation that various lines can simultaneously occur and influence each other. Illness work can be facilitated as users of online support groups share links to preferred exercise and nutrition resources such as calorie counters and offer tips for managing illness issues. For example, one community member offers ideas for activities and connects those tips to a larger resource:

Swimming is a great exercise and can help you lose weight. :-) In fact, there are lots of small things you do everyday (walking for instance) that help burn calories and thus weight…You can do it though, just don’t give up! If you are interested, The American Cancer Society just made this wellness type program for women, and you can choose a weight goal and people can follow you. Like I said, don’t know if you are interested, but thought I would share. :-D

Everyday life work coincides with communication work when digital communication tools aid management of food-related concerns, such as recipe-planning. Using digital tools to share information sources about weight and exercise also overlaps with biographical work by impacting self-perceptions of identity. A member discusses how a communication technology—a phone application, in this case—helped with the everyday effort of food planning and eventually aided biographical development as this individual experienced success with weight management:

I’ve got a great app on my phone called Lose It, which I use to track my calories. I’ve told it that I want to lose two lbs a week, and it gives me a calorie limit per day. I track everything I eat and when you go over your daily limit, the chart turns red. The program has a huge database of food (calorie counts for many restaurants). It can be difficult to get into the habit, but now I’m addicted to tracking calories for everything.

Communication work, biographical work, and everyday life work co-mingle for this individual to drive better outcomes for weight management and body composition goals. Communication tools help manage the actual everyday task of food preparation toward the end goal of a different leaner physical self-presentation.

Communication work stands out as a varied but essential element of young adults’ illness trajectories, including how they design messages, manage information, and employ technology. Communication work also intersects with other kinds of work related to exercise and nutrition, including illness work through tips on symptom management, everyday life work involving recipe-sharing or meal-planning, and biographical work in helping plan messages to discuss sensitive issues, to name some examples.

## Discussion

### Principal Results

The findings of this article, in response to our first research question, describe how young adults affected by cancer experience significant exercise and nutrition needs and the notion of work helps to define their efforts, as these individuals manage the illness, everyday life, biographical, and communication aspects of cancer [13,15]. The four lines of work overlap and compete for limited resources during the time when young adults report significant unmet needs related to exercise and nutrition and strive to create a “new normal” for themselves [2].

A more nuanced grasp of how young adults deal with exercise and nutrition can help develop support efforts and approach the well-documented psychosocial challenges particular to cancer diagnoses during their life stage [2,8,70]. Nurses, dietitians, social workers, and health communication practitioners, for example, could use these findings to tailor support efforts, focusing on the ways young adults affected by cancer balance competing work demands across varying illness trajectories.

This study also expands the demographic focus of illness trajectory research by examining the young adult experience, whereas other illness trajectories research has tended to involve middle-aged or older participants. Such expansion helps better document the understanding of illness trajectories and allows for comparison among age cohorts.

Additionally, driven by our second research question, this article extends the concept of communication work into the realm of digital media and online support. The study expands the application of research into the interplay among communication channels to examine the mix of interpersonal and mediated communication, building upon information-flow and limited-effects streams of research [71,72]; individuals undertake communication work by moving back and forth between online and offline contexts. Young adults in this sample participate in person-to-person conversations through posts in the online group about exercise and nutrition and then use those interactions to shape face-to-face conversations with friends and family. Young adults can also use older posts in the support group as an archive to gather needed content for face-to-face connections with loved ones and care providers—occasionally re-starting long-dormant online conversation threads out of a need for further information, support, or interaction. The result is an understudied “mass personal” mix of communication channels bringing the digital and the physical together into an essential resource for young adults laboring to complete work.

Understanding this interplay of communication channels offers opportunity to create focused interventions based upon the way individuals prefer to use media as well as their information needs [73]. Previously tested health interventions tailored to cultural norms, health literacy, and media-use preferences have demonstrated the impact and importance of fully considering audience factors [74,75]. Taking advantage of channel preferences or sharing across channels can be equally important. If the desired audience prefers to receive new information through short Facebook posts so that the knowledge can be discussed at more length face-to-face among friends and then further spread through crystalized 140-character Twitter messages, this is the scenario for which health promotions professionals should prepare. In other health contexts, patients have reported appreciating when health care professionals express openness to patients pursuing and discussing outside information [76].

Food- and activity-related tasks become required, demanding, and effortful while needing coordination and management over time for the expectation of longer-term benefits. Our findings indicate that illness, everyday life, biographical, and communication work concerning exercise and nutrition are relevant to this population in different but overlapping ways. Illness work is expressed mostly around modifying activity and diet to fight cancer itself as well as making changes to manage side effects. Young adults discuss illness work related to exercise and nutrition as a means of maximizing health during difficult and stressful times.

Exercise and nutrition’s role in everyday life work for young adults comes out as onerous and particularly demanding because of physical and emotional limitations from treatment as well as the barrier of the biographical-work comparison to their pre-cancer selves. Young adults discuss the need to re-start exercise regimens as a source of significant frustration, and the same applies to nutritional changes. Community members discuss feeling forced to simultaneously manage the work of deliberately shifting food-consumption patterns while grappling with body-image issues related to weight gain. Such feelings indicate an area for nursing or social work support to overcome challenges common to young adult nutrition and exercise concerns [77]. Support services focused on helping young adults affected by cancer manage needed nutrition changes can help routinize and ease the burden of practical barriers such as learning to cook.

Biographical work stands out as the most discussed area of work within our data, as it is both prominent on its own and frequently woven into other lines of work while young adults struggle to manage body-image and self-esteem questions. Key identity re-construction takes place here in a time-related manner [34]. Many individuals frame identities through past exercise and nutrition experiences while ongoing movement and food challenges strongly affect current self-perceptions. Looking to the future, young adults use exercise and nutrition as a tool to actively build hope into their expectations by employing activity and diet to fight off future problems and maintain wellness. Knowledge of the food and exercise behaviors that help young adults feel empowered relating to their futures could play a useful role in supportive educational interventions, where the need for nutritionally focused efforts is particularly acute. Little research has examined nutritional/dietary interventions for cancer survivors in general [78], and almost none looks into young adults specifically [77]. This line of research into nutritionally influenced health and identity outcomes presents significant potential for impact on young adults’ cancer trajectories.

Communication work plays an involved and nuanced role in how young adults express, share, and manage exercise and nutrition across illness trajectories. Users of the online community studied here employ a “mass personal” approach where they work seamlessly between their face-to-face conversations and online interactions to gather information, discuss thoughts and feelings, and prepare for interpersonal conversations of all kinds, including elsewhere online, with medical professionals, and with colleagues. Such communication patterns open opportunities for the digitally focused support interventions shown to be effective for mental and physical well-being of individuals dealing with chronic illness such as cancer [79].

Young adults often complete communication work in a range of ways to relate exercise and nutrition experiences to others, and these efforts intersect with other lines of work, as expected from prior research. Communication work on- and offline helps individuals manage preparations for message-design activities needed to complete illness work related, for example, to explaining diagnoses to others and biographical work about expressing and understanding the cancer experience. Everyday life work involves communication work when young adults employ communication tools such as online social network and phone applications to manage knowledge about exercise plans or to track nutrition planning. Health professionals would be well served to consider the interplay and range of work demands and communication channels when interacting with patients. By understanding how and why young adults employ sources and content, care providers can proactively address questions and offer accurate, useful content in the ways their patients prefer and are more likely to use.

### Limitations

In line with the contributions of this research, it is also essential to note some limitations. Our dataset, being de-identified text from years of online conversations, was already established, eliminating the ability to directly probe at specific items. Reported behaviors and examples of work were thus not verifiable. Further, certain economic and cultural groups are less likely to use such resources [80], and it is also likely that certain treatment centers encourage online support more than others. As our sample consisted of de-identified text, demographic information could not be collected. The online community studied here includes individuals from a broad, English-speaking geographic range spanning ages 18-39 and dealing with a number of cancer diagnoses; thus, the data are useful for young adults affected by cancer but cannot effectively be applied to specific conditions, places along the cancer trajectory, or subgroups. Also, supporters of those diagnosed with cancer were not present in the data and could offer further depth and perspective on the young adult cancer experience related to illness trajectory work and “mass personal” communication. More research through different methodologies and topic areas beyond exercise and nutrition is needed to examine the role of work among young adults.

### Comparison With Prior Work

Examining exercise and nutrition within the experience of young adult cancer makes several theoretical contributions when compared to prior research. First, this research brings the theory of illness trajectories into a new demographic and validates key concepts in a different sample. The demographic expansion adds nuance to our understanding of work, particularly biographical work, by studying its impact among individuals in a different life stage than older populations previously studied. As discussed earlier, young adults are more likely to use technology, be in a transitive life stage, have particular information needs, and actively deal with age-specific psychosocial needs. All of these factors help design tailored intervention messages to reach young adults with relevant information.

Second, the contents of this article help validate the recent communication work addition to theory of illness trajectories research [13,15] while extending the communication work addition to a mediated context. Our analysis demonstrated that communication work was present throughout the data. The fact that the conversations we analyzed were natural and unprompted, yet full of examples of communication work, lends credibility to the premise that communication work is a part of living with cancer. Understanding the presence of communication work can help support personnel better prepare those affected by cancer for the effort required.

Additionally, we examine the interplay of interpersonal and mediated communication in a “mass personal” environment where individuals complete communication work by co-mingling online and offline worlds. Community members in this sample create person-to-person interactions by exchanging posts in the online group about exercise and nutrition and then complete face-to-face conversations based on the experience of the online interaction. Older conversations serve an archival purpose useful for message preparation activities, resulting in a mix of communication channels that brings the online and offline together.

More practically, this article also adds to understanding of how food and activity affect the young adult cancer experience and also how young adults complete work to manage exercise and nutrition needs. Using young adults’ own words allows for a more thorough understanding about how they discuss this top-level concern, which in turn sets up opportunities for more effective interventions. Furthermore, a better grasp of how young adults affected by cancer use different types of media to complete lines of work aids in planning for how to best help them finish those tasks. With knowledge about how they approach work, it becomes possible to train and coach young adults to more effectively balance the requirements of illness and biographical work, for example.

### Conclusions

This article adds to our understanding of how young adults affected by cancer deal with one of their primary unmet needs, exercise and nutrition information. By applying the valuable heuristic concepts of illness, everyday life, biographical, and communication work, we have improved understanding of the young adult cancer experience and described processes by which they manage a series of challenges inherent in a cancer diagnosis to create a “new normal” [2]. A more nuanced understanding of how young adults handle exercise and nutrition can help provide support and make progress with the well-documented psychosocial challenges particular to this demographic [2,8,34], including re-developing a personal identity, maintaining a positive outlook on the future, maximizing health outcomes, and managing communication demands.

The “mass personal” way in which the young adults in this support community move between communication channels allows them to complete work in a number of ways, particularly relating to learning about exercise and nutrition needs as well as expressing their dietary limits. Members in the community gather information from mass media and interpersonal conversations with health care providers before turning to online groups to make sense of that content. Active and archived online group discussions additionally help users complete the work involved with processing their experiences and preparing for upcoming face-to-face and social media interactions. Traditional mass media blur with typically interpersonal outlets in a number of ways that allow for these young adults to create more personalized communication messages that can be delivered to one person or many and receive feedback on that content from the same, potentially long after the initial interaction.

Further investigation of communication patterns and lines of work in the illness trajectories of young adults affected by cancer could lead to interventions that improve on their comparatively poor health and psychosocial outcomes [1,2], including acute need for exercise and nutrition information [5,7].
